# External Evaluation of Population Pharmacokinetics Models of Lithium in the Bipolar Population

**DOI:** 10.3390/ph16111627

**Published:** 2023-11-18

**Authors:** Lereclus Aurélie, Boniffay Andréa, Kallée Gauvind, Blin Olivier, Belzeaux Raoul, Frédéric Dayan, Benito Sylvain, Guilhaumou Romain

**Affiliations:** 1 Institut de Neurosciences des Systèmes, Aix Marseille Université, Inserm UMR 1106, 13385 Marseille, Franceromain.guilhaumou@ap-hm.fr (G.R.); 2 EXACTCURE, 06000 Nice, Francef.dayan@exactcure.com (F.D.);; 3 Service de Pharmacologie Clinique et Pharmacovigilance, Hôpital de la Timone, 13005 Marseille, France; 4 Pôle Universitaire de Psychiatrie, CHU de Montpellier, 34000 Montpellier, France

**Keywords:** lithium, bipolar disorder, population pharmacokinetics, external evaluation, therapeutic drug monitoring, treatment optimization

## Abstract

Lithium has been used in the treatment of bipolar disorder for several decades. Treatment optimization is recommended for this drug, due to its narrow therapeutic range and a large pharmacokinetics (PK) variability. In addition to therapeutic drug monitoring, attempts have been made to predict individual lithium doses using population pharmacokinetics (popPK) models. This study aims to assess the clinical applicability of published lithium popPK models by testing their predictive performance on two different external datasets. Available PopPK models were identified and their predictive performance was determined using a clinical dataset (46 patients/samples) and the literature dataset (89 patients/samples). The median prediction error (PE) and median absolute PE were used to assess bias and inaccuracy. The potential factors influencing model predictability were also investigated, and the results of both external evaluations compared. Only one model met the acceptability criteria for both datasets. Overall, there was a lack of predictability of models; median PE and median absolute PE, respectively, ranged from −6.6% to 111.2% and from 24.4% to 111.2% for the literature dataset, and from −4.5% to 137.6% and from 24.9% to 137.6% for the clinical dataset. Most models underpredicted the observed concentrations (7 out of 10 models presented a negative bias). Renal status was included as a covariate of lithium’s clearance in only two models. To conclude, most of lithium’s PopPK models had limited predictive performances related to the absence of covariates of interest included, such as renal status. A solution to this problem could be to improve the models with methodologies such as metamodeling. This could be useful in the perspective of model-informed precision dosing.

## 1. Introduction

Bipolar disorder (BD) is a serious mood disorder that affects up to 4% of the world’s population and is among the leading causes of disability [1]. It is characterized by recurrent episodes of depression alternating with episodes of hypomania and/or mania that are usually separated by periods of relatively normal mood and functioning [2]. Pharmacological treatment is extremely important for managing patients with BD. During acute episodes, treatments are used to reduce symptoms and hopefully to obtain a full remission. For maintenance treatment, drugs are used to prevent the recurrences of mood episodes [3].

Lithium was the first drug to be effective in the treatment of BD, and has been used for over sixty years [4]. It is, indeed, the oldest and most prescribed drug in this pathology, and it presents a high response rate [5,6,7]. Lithium’s mechanism of action in mood regulation is not entirely clear, but is progressively being understood and involves the direct inhibition of glycogen synthase kinase 3β and various effects on neurotrophic factors, neurotransmitters, oxidative metabolism, apoptosis, second messenger systems, and biological systems [4]. Concerning its pharmacokinetic (PK) properties, this drug is not metabolized, and is almost exclusively excreted via the kidney in the form of a free ion. It is able to freely cross the glomerular membrane and is reabsorbed at 80% via passive diffusion in the proximal tubules [8]. This makes renal status, and thus the glomerular filtration rate (GFR) or creatinine concentration and clearance, important predictors of lithium’s elimination [1]. In addition, the reabsorption of lithium is competitively inhibited by sodium; indeed, sodium deficiency has proven to promote lithium reabsorption and then induce an increase in serum lithium concentration [9]. It has also been found that body size could be a predictor of lithium’s clearance [10].

Though lithium is an efficient drug in BD, its clinical use remains complex due to its PK and pharmacodynamic (PD) properties. Indeed, lithium has a narrow therapeutic range (0.4–0.8 mEq/L, up to 1.2 mEq/L un acute phase), and displays significant intra-patient PK variability [8,11]. Therefore, predicting the right dose for each patient remains challenging, and treatment optimization is needed for this drug. An overexposure to lithium can induce progressive or acute renal insufficiency and adverse events such as nausea, vomiting and diarrhea. To ensure safety and avoid toxic adverse events, international guidelines recommend the close monitoring of concentrations through therapeutic drug monitoring (TDM) [12], even though studies have found that the prevalence of TDM for lithium was low [13,14]. 

In addition to TDM, attempts have been made to predict adapted lithium doses to attain therapeutic levels, and one of them relies on population pharmacokinetics (popPK) models to individualize dosing regimens [15]. Indeed, modeling and simulation methods have shown major advantages in several therapeutic areas in supporting dosing regimen selection for patients, like in the dosing adjustment of antibiotics [16,17]. In the psychiatric field, numerous molecules require precision dosing; however, we observed that only few popPK models of psychotropic drugs have been developed, even for lithium, which has been used for several decades. Moreover, prior to any kind of use for model-informed precision dosing (MIPD), the predictability of the models must be assessed. Indeed, the external evaluation of models has proven to be an important additional feature and to be necessary for use in patients’ dose individualization in clinical settings [18]. However, few popPK models developed in psychiatry have been externally evaluated. Among the popPK models of lithium identified in the literature, only one used external evaluation to assess model predictive performance.

Therefore, this study aimed to assess the applicability for MIPD of previously published popPK models of lithium by testing their predictive performances. Two different external datasets were used including clinical or literature data.

## 2. Results

### 2.1. Published Models

**Figure 1:**
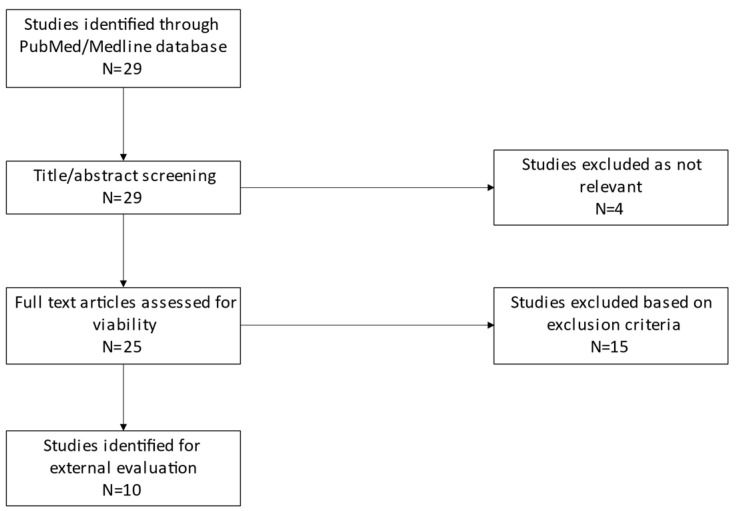
Overview of the strategy used in the literature search for PopPK models of lithium. N: number of articles returned by the search.

After the literature search, 10 popPK models were identified [10,19,20,21,22,23,24,25,26,27] (see Figure 1). Three studies were conducted in China and two were conducted in the USA. The other studies were conducted in Japan, Egypt, China, France, Thailand and Saudi Arabia (Table 1). Only two studies included more than 100 subjects [10,26]. All models followed linear first-order kinetics. Lithium’s PK was described using one-compartmental models in three studies and two-compartmental models in seven studies. In those compartmental models, the absorption phase was described in eight models by a fixed constant of absorption (ka). The volume of distribution (V) was fixed in three models, unlike clearance (CL), for which inter-individual variability was estimated in all the models. Overall, 11 different covariates were tested in the 8 published models (weight, age, gender, serum creatinine (Scr), glomerular filtration rate (GFR), creatinine clearance (CrCl), body mass index (BMI), co-administration (risperidone, diazepam, clonazepam, valproic acid, and perphenazine), lean body weight (LBW), fat-free mass (FFM), and total daily dose (TDD) and 7 were included in parameter estimation: FFM (n = 1), LBW (n = 1), GFR (n = 1), weight (n = 4), age (n = 1), ClCr (n = 2), and TDD (n = 1). FFM was included for V1, V2 and CL in one study. LBW was included for CL, V1, V2 and Q in one study. Weight was included for V1 in two studies, for CL in three studies and for V2 in one study. GFR, age and TDD were included for CL in one study each. And ClCr was included for CL in two studies.

### 2.2. External Evaluation Data

**Figure 2:**
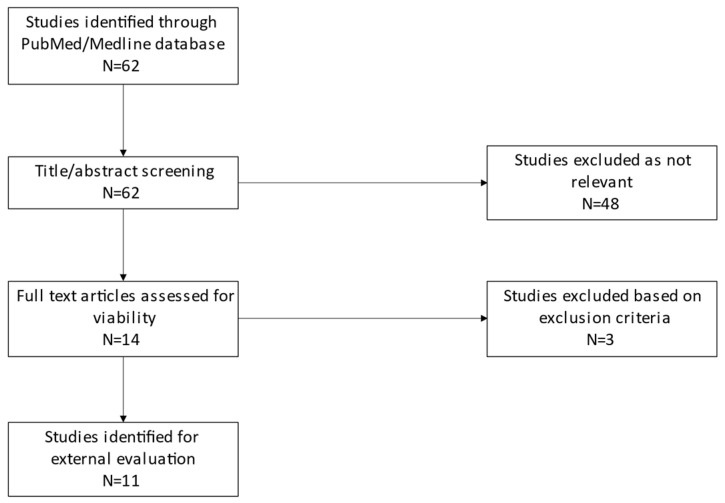
Overview of the strategy used in the literature search for individual PK data of lithium. N: number of articles returned by the search.

The literature dataset was composed of 89 concentration data samples from 89 patients (62 males and 27 females) in 11 different studies (see Figure 2). Concentration data were provided from single-dose studies in healthy volunteers or PK studies of bipolar patients in a steady state and corresponded to through concentration or Cmax. The exact time of the sample was not available. The characteristics of the datasets are summarized in Table 2. We observed a significant variability, with patients’ presented age ranging from 8 to 87 years old and weight ranging from 27 to 125 kg. Amongst them, GFR was not described for 50 patients. For the 39 others, the mean GFR value was 120.61 (± 88.54) mL/min. 

The clinical dataset was composed of 46 concentration data samples from 46 patients (20 males and 26 females). All samples were through concentrations at steady state, and the exact time at which the sample was taken was available. The demographic data are summarized in Table 2. Patients had ages ranging from 16 to 68 years old and weight ranging from 49 to 125 kg. For each patient, GFR was reported for all 46 patients, and ranged from 61 to 127 mL/min. 

### 2.3. External Evaluation

**Figure 3:**
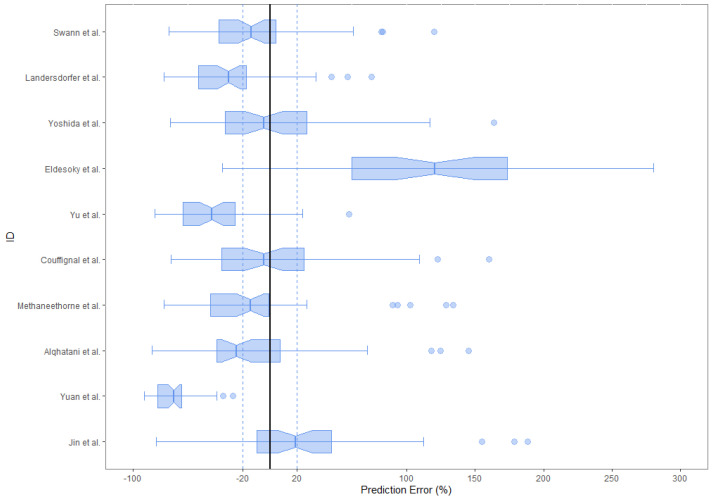
Box plots of the PEs for the 10 published popPK models for the clinical dataset. The black solid lines and blue dotted lines are reference lines indicating the PE% of 0% and ±20%, respectively.

**Figure 4:**
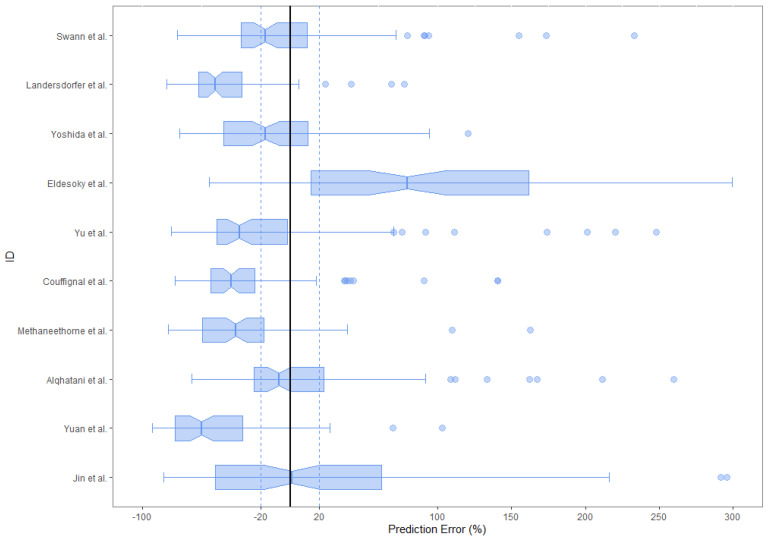
Box plots of the PEs for the 10 published popPK models for the literature dataset. The black solid lines and blue dotted lines are reference lines indicating the PE% of 0% and ±20%, respectively.

The results of the external evaluation are presented in Table 3. All the samples from both datasets were used to evaluate the predictive performance of all models. For the literature dataset, the MDPE ranged from −6.6% to 111.2% and the MADPE ranged from 24.4% to 111.2%. Only two models, the ones by Alqahtani et al. and by Swann et al., met the aforementioned criteria (MDPE ≤ ±20% and MADPE ≤30%) [19,25]. For the clinical dataset, the MDPE ranged from −4.5% to 137.6% and the MADPE ranged from 24.9% to 137.6%. Only three models, the ones by Swann et al., Jin et al. and Methaneethorn et al., met the aforementioned criteria [19,26,28]. Only the model by Swann et al. met the acceptability criteria for both datasets [19]. Most models had an MDPE ≤ 40% and MADPE ≤ 50%. Figure 3 and Figure 4 show the boxplots of the PE for all 10 published models for each dataset.

**Figure 5:**
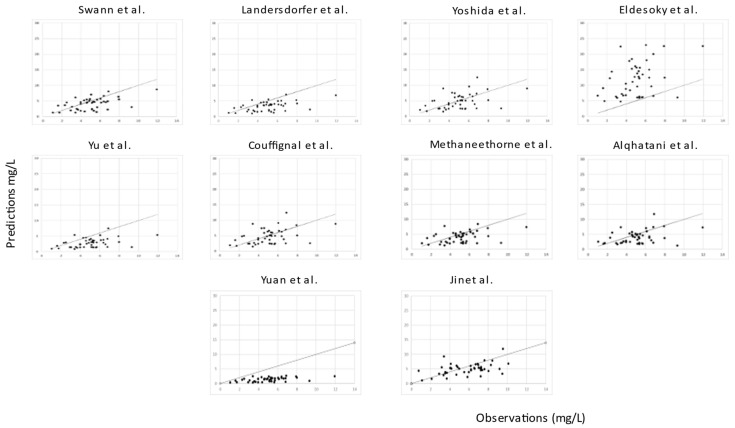
Predictions (mg/L) versus observations (mg/L) of the 10 published popPK models for the clinical dataset.

**Figure 6:**
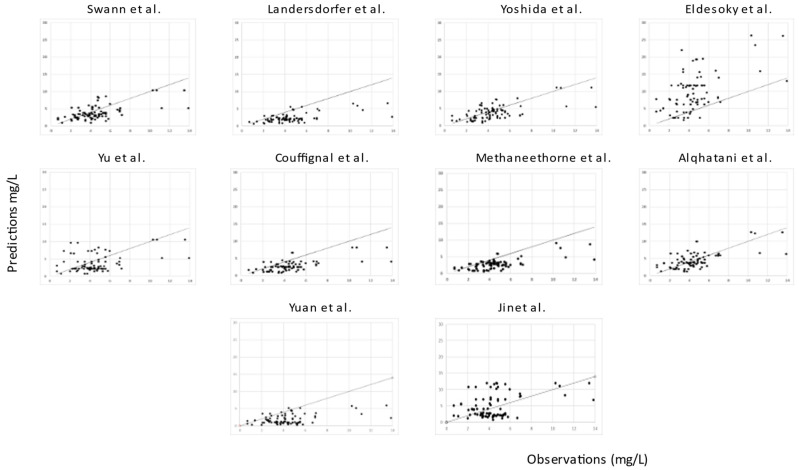
Predictions (mg/L) versus observations (mg/L) of the 10 published popPK models for the literature dataset.

**Figure 7:**
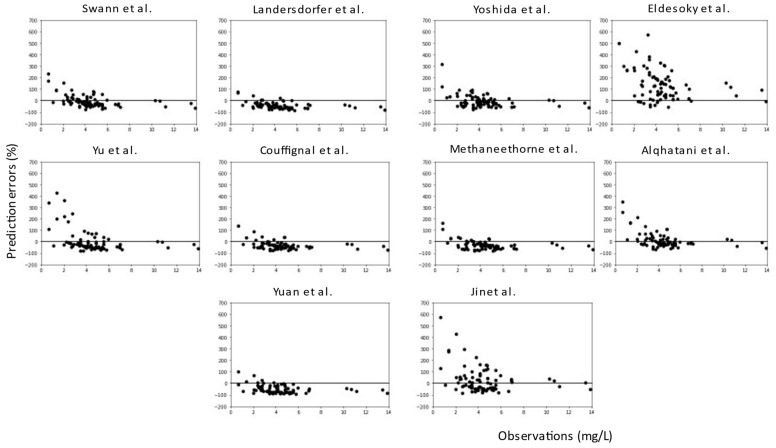
Prediction errors (%) versus observations (mg/L) of the 10 published popPK models for the literature dataset.

**Figure 8:**
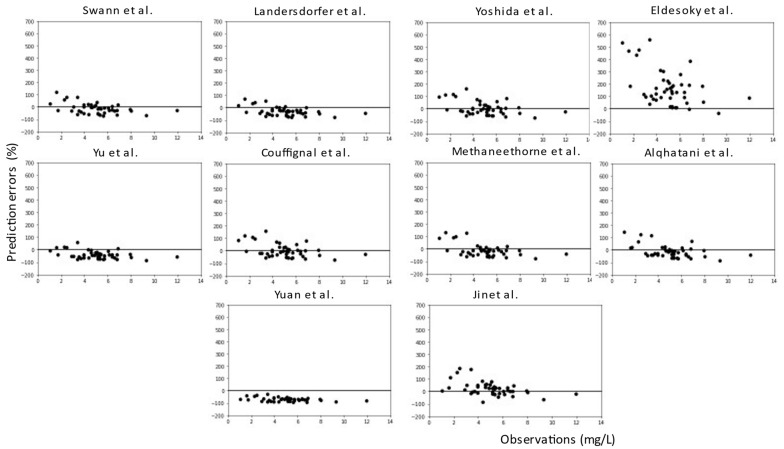
Prediction errors (%) versus observations (mg/L) of the 10 published popPK models for the clinical dataset.

For all models, predictions were plotted versus observations for each dataset (see Figure 5 and Figure 6). Overall, most models had a tendency to underpredict the concentrations. For both the literature and the clinical dataset evaluation, 7 out of 10 models had a negative bias. For the 10 models, the PEs were plotted versus observations to visualize whether there was a difference in the predictive performance according to concentration (see Figure 7 and Figure 8). We observed a higher inaccuracy in the low-concentration predictions.

To assess the impact of patients’ renal status on the models’ predictive performances, the PEs were plotted versus GFR. No relationship between GFR and PE could be seen (see Supplementary Figures S1 and S2). 

## 3. Discussion

In a recent systematic review for popPK models of lithium, the authors point out the lack of data on the evaluation of predicate performance of these models [10]. From the perspective of the model-based personalization of lithium therapy, we conducted an external evaluation of published lithium popPK models. To the best of our knowledge, this is the first study that evaluated the predictive performance of popPK models of lithium using independent external evaluation datasets. External evaluations are usually conducted in clinical datasets from hospitalized patients. However, obtaining a qualitative clinical dataset of concentrations for lithium is quite difficult due to the characteristics of the studied population; many of them were outpatients, meaning it was difficult to obtain robust information on the time of the last drug intake and there was possibly poor adherence to medication [29]. Consequently, we tested an alternative method using literature-extracted individual data as an external evaluation dataset, and we compared the results to an external evaluation for clinical data. 

During our evaluation, significant variability was observed in the predictive performance of the studied popPK models, and most of them presented limited results. Moreover, we observed a negative bias trend, concentrations being mostly underpredicted. This could be due to a lack of representation of some significant causes of variability for lithium’s elimination, such as sodium concentration or the coadministration of other drugs, which might have an impact on the renal elimination of lithium [9]. 

Among the available popPK models of lithium, only one met the acceptability criteria for each dataset [19]. This model, by Swann et al., included FFM as a covariate, and was slightly better for the clinical dataset for which this covariate was fixed for none of the patients. The model by Alqhatani et al. [30] presented the best predictive performance for the literature dataset, but did not meet the acceptability criteria for the clinical dataset. This model included renal status as a covariate, and therefore presented better performance for patients with variable renal function (renal impairment or augmented renal clearance), as observed in the literature dataset. Two models, the one by Jin et al. [26] that included CrCl, weight and TDD as covariates, and the one by Methaneethorne et al. [28] that included weight and age as covariates, presented good predicting performances for the clinical dataset but not for the literature dataset. This could be explained for both models by the higher variability of patients’ characteristics observed in the literature dataset, which includes different populations from different studies and therefore generates a more heterogenous dataset. Moreover, in the clinical dataset, covariates’ information was available for the whole population, whereas they were fixed for some of the patients from the literature dataset. Another example of the heterogeneity of the literature dataset is the analytical methods of lithium quantification. Different methods were observed from one study to another, and could also partly explain the differences in predictive performances for both datasets [31]. 

Two models presented poor predictive performances for both datasets. These results were expected for the model by Yuan et al. [27], as it was built on a pediatric population with a different pathology and therefore did not fit our populations. The model by Eldesoky et al. [22] was the least predictive for both datasets. These results are consistent with the population prediction versus observation graphs observed in this study, which show the low predictability of the model. However, the individual prediction versus observation graph of this model shows a better predictability, suggesting that approaches such as Bayesian forecasting could improve the results of the external evaluation of this model. Unfortunately, we could not perform Bayesian forecasting in this study, as only one sample per patient was available in both datasets.

The studied covariates can be classified in two categories: the ones that impact the elimination of the drug (GFR, ClCr, and age), which were included in three models [22,26,30], and the ones that impact the distribution (weight, FFM, LBW, and body size), which were included in six models [19,21,26,27,28,32]. However, most models failed to include those covariates alone or together, and, most significantly, failed to include the impact of renal status on lithium clearance. For example, the most predictive model [19] failed to include renal status as a covariate of lithium’s elimination, even though it is well known that lithium’s elimination is mainly renal (90%). This issue is probably due to the lack of variability of this covariate in their training dataset. For patients with normal renal function, this model would be efficient; however, it would fail to include the variability expected for lithium elimination in patients with altered renal function. Moreover, we observed that models including covariates impacting the elimination of the drug tended to present better predictive performances for the literature dataset, and models including covariates impacting the distribution of the drug tended to present better predictive performance for the clinical dataset. These results could be due to the significant variability of renal status observed for the literature dataset and the fact that weight, age, and height were not available for a significant number of patients in the literature dataset. 

Studying and comparing the details of the predictions for both datasets, overall, we observed qualitatively similar results. Therefore, our study supports that a literature dataset could be an acceptable alternative in PopPK external evaluation procedures, but requires data imputation for coping with missing covariates [33,34]. These results seem interesting, because the main challenge for the external evaluation of models remains the ability to gather enough reliable retrospective data to perform the analysis [35]. This methodology can be an interesting option for drugs such as lithium, for which data are hardly available or often not of good quality, as most patients are outpatients (lack of demographic information and potential noncompliance to treatment). In addition, gathering clinical data is time-consuming and challenging; it requires either a dedicated prospective study or retrospective data extraction cleaning in hospital information systems, and data reliability is not always guaranteed. There are, however, limits to this methodology. In our literature dataset, we had access to a limited variability of endpoints of interest (peak/trough concentration), and we had no choice in the population in which the data were available. In addition, all the necessary covariates were not systematically available for each patient. Therefore, we had to perform a simple data imputation by fixing some of them to a standard value, thus losing patient-centered information to provide to the models. Indeed, in the literature dataset, only the described covariates could be used, whereas in a clinical dataset we can prospectively gather all the needed covariates. This limitation probably explains the better results of the external evaluation of the clinical dataset than the literature dataset in 6 out of the 10 models. 

## 4. Materials and Methods

### 4.1. Search Methods and Inclusion Criteria

We performed a literature search in PubMed/Medline/Prospero/Clinicaltrials (accessed on 31 May 2023) for all popPK models of lithium published before December 2022. The used query was “lithium [Ti] AND (population pharmacokinetics OR model)”. To be included in this review, the study had to meet the following inclusion criteria: (1) models had to be popPK models of lithium, (2) all the parameters (fixed effects) of the model had to be explicitly described, such as all the population equations, and, (3) the publication had to be written in English. Exclusion criteria were if the study were a review, on animals, or in vitro.

### 4.2. External Evaluation Data

#### 4.2.1. Literature Cohort

We performed a literature search in PubMed/Medline/Prospero/Clinicaltrials (accessed on 31 May 2023) for all individual pharmacokinetics or blood samples of lithium published before December 2022. The used query was “lithium [Ti] AND (pharmacokinetics)”. All sources describing popPK models identified from the first query were excluded. To be included in our dataset for external evaluation, the study had to meet the following inclusion criteria: (1) the study had to contain data associating plasma lithium concentration with each patient, a time of sampling, a dosing regimen of lithium and descriptive information of the patient, and (2) the publication had to be written in English. Exclusion criteria were if the study were a review, conducted on animals, or in vitro. Among the covariates of interest that were used by at least one of the models, the ones for which the values were not available in the study for each individual were fixed to the population mean of the study or to a standard value if the information was not available. 

#### 4.2.2. Clinical Cohort

Data were retrospectively collected from patients with BD followed in the FondaMental Bipolar Disorder Expert Center of Marseille from the 11 September 2022 to the 5 December 2022. This study was approved by the Marseille university hospital committee as a retrospective, noninterventional, and anonymous study, following the European GDPR standards (RGPD/AP-HM N8 2021-85). Patients included in this study were treated with lithium and received at least one dose of lithium for TDM. To ensure the robustness of the data and pharmacological data (sample time, time of last dose, and dosing regimen), demographic and physiological data (age, weight, glomerular filtration rate, sex, and height) were collected afterwards from the patients’ files. The time and dosing regimen were precisely collected. The exact time of blood sampling was recorded by the attending medical staff. Lithium concentrations were analyzed via colorimetric assays using a Cobas 8000 analyzer (Roche Diagnostics^®^) between the 11 September 2022 and the 30 May 2022 and using an AtelliCha analyzer (Siemens^®^) between 30 May 2022 and 5 December 2022. Accuracy between the two methods was guaranteed with a cross validation study and when a mean bias <10% was observed. The limit of quantification was 0.1 mmol/L for the two methods.

### 4.3. Model Evaluation

The external evaluation was conducted using the ExactCure simulation library, which is developed in Python language (European Single Registration number: FR-MF-000003719). The final popPK models were simulated based on the formulas and parameters reported for each of the included popPK studies. The external evaluation was performed without any additional fitting of the model to the data.

The prediction error (*PE*) and absolute prediction error (*APE*) were used to assess the predictive performance of the models. *PE* and *APE* were determined using the following equations:

$$PE\left(\%\right)=\frac{Cpred-Cobs}{Cobs}\times 100$$

$$APE\;\left(\%\right)=\left|PE\right|\times 100$$

where *C_pred_* and *C_obs_* are the predicted and observed concentration, respectively.

The median prediction error (*MDPE*) and the median absolute prediction error (*MADPE*) were used as a measure of bias and inaccuracy, respectively.

1. Bias (i.e., the direction and the size of the deviation from the observed concentration) of model predictions is given for the *i*th individual and th *j*th blood sample, by the median prediction error (*MDPE*):
  $$MDPE(\%)=MEDIAN\left(PE(\%)\left(ij\right)\right)$$
2. Inaccuracy (i.e., the size of the typical error) of model predictions is given for the *i*th individual and the *j*th blood sample, by the median absolute performance errors (*MADPE*).
  (1) $$MADPE(\%)=MEDIAN\left(APE(\%)\left(ij\right)\right)$$

A MDPE between −20% and 20% and a *MADPE* ≤ 30% are considered to be acceptable criteria for bias and inaccuracy [36]. 

## 5. Conclusions

To conclude, published lithium popPK models were evaluated using two different datasets, one from clinical data and one from literature data. Our study showed that gathering an external evaluation dataset from the literature could be an alternative when clinical data are not readily available. In addition, even if clinical data are available, both approaches could be used in combination to increase the data and therefore the reliability of the external evaluation. We observed a heterogeneity in the predictive performances of the models, and only one model met the acceptability criteria for both datasets and included FFM as a covariate. This model could be used in clinical practice for lithium to provide a supportive tool to determine the optimal dose, and then to assist drug dosage decisions during treatment titration. However, it failed to incorporate renal status as a covariate of lithium’s elimination. Methodologies like metamodeling, which allow the amalgamation of different models’ information into one meta model, could allow a more predictive model, including both covariates of interest from the perspective of MIPD, to be built for this drug.

## Figures and Tables

**Table 1:** Literature review summary.

Study (Publication Year)	Country (Single/Multiple Sites)	Number of Patients (Male/Female)	Number of Samples (Sample Type)	Analytical Method	Pathology	Software	Number of Compartments of the Model	Covariates Tested	PK Parameters and Formula (Lithium)		IIV (%)	Residual Error
Swann et al. (1990)	USA (single)	14 (10/4)	NA (pk study)	Atomic absorption spectrometry	Manic episode	None	Two	FFM	CL (L/h) V1 (L) V2 (L) K12 (h^−1^) K21 (h^−1^) Ka * (h^−1^)	0.09 × V1 0.3 × FFM (0.4 × FFM)−0.3 0.56 0.41 1.03		
Landersdorfer et al. (2016)	USA (multiple)	61 (32/29)	2730 (pk study)	Atomic absorption spectrometry, ICP-OES, automated assays	Bipolar disorder	S-ADAPT/ NONMEM	Two	TBW, age, LBW, gender, CrCL, Scr	CL (L/h) V1 (L) V2 (L) Q (L/h) Klag (h^−1^) Ka (h^−1^)	1.63 ×(LBW/53)^0.75^ 24.5 × (LBW/53) 33.5 × (LBW/53) 3.19 × (LBW/53)^0.75^ 3.64 1.21	20.7 11.6 40.3 31.1 88.2 21.0	0.0422 mEq/L
Yoshida et al. (2017)	Japan (multiple)	82 (44/38)	131 (TDM)	Atomic absorption spectrometry	Bipolar disorder, depression, schizophrenia	SPSS/PRISM	One		CL (L/h) Ka * (h^−1^) V1 * (L)	1.236 1.5 0.79 × WT		0.13 mEq/L
Eldesoky et al. (2008)	Egypt (single)	50 (21/29)	50 (TDM)	Ion sensitive electrode	Bipolar disorder	NONMEM	Two	WT, GFR, gender, age	CL (L/h) V1 * (L) V2 * (L) Q * (L/h) Ka * (h^−1^)	0.51 × (GFR/105.3)^0.438^ 15.2 7.44 6.7 0.62	12.77	15%
Yu et al. (2016)	China (single)	20 (20/0)	560 (pk study)	Flame photometry	Healthy	NONMEM	Two	Demographic factors and laboratory indicators	CL (L/h) V1 (L) V2 (L) Q (L/h) Tlag (h) Ka * (h^−1^)	9.39 10.4 216 22.1 0.186 0.293	13.2 84.2 19.7 127 19.4	5.19%
Couffignal et al. (2018)	France (single)	17 (6/11)	68 (pk study)	ICP-OES	Bipolar disorder	MONOLIX	Two	None	CL (L/h) V1 (L) V2 * (L) K12 * (h^−1^) K21 (h^−1^) Tlag * (h) Ka * (h^−1^) F	1.21 23 64.7 3.63 9.46 0.92 2.22 0.62	20 30 27 72 0.98	10%
Methaneethorne et al. (2019)	Thailand (single)	222 (114/108)	NA	Ion-sensitive electrode	Bipolar disorder	NONMEM	One	Weight, age, gender, Scr, CrCl, BMI, co administration.	CL (L/h) Ka * (h^−1^) V1/F * (L)	1.43 × (WT/65)^0.425^ × (age/38)^−0.242^ 0.462 54	3.11	19 mg/L
Alqahtani et al. (2019)	Saudi Arabia (single)	31 (7/24)	170 (TDM)	Spectrophotometric methods	Bipolar disorder	MONOLIX	Two	CrCl	CL (L/h) V1 * (L) V2 * (L) Q * (L/h) Ka * (h^−1^)	1.15 × (ClCr/119.2)^0.117^ 22.1 3.35 0.44 0.62	14	0.223 mmol/L
Yuan et al. (2021)	China (single)	52 (38/14)	160 (pk study)	Ion sensitive electrode methos	Intellectual disability, pediatric	NONMEM	Two	Weight, age, gender	Cl/F (L/h) MTT(h) V1/F (L) V2/F (L) Q/F (L/h)	0.98 × (WT/20) 0.52 13.1 × (WT/20) 8.2 × (WT/20) 0.84	4.6 9.9 7.2 17.7 9.5	0.091 mmol/L
Jin et al. (2022)	China (single)	268 (NA)	476	Phosphatase assay (Siemens^®^ ADVIA 1800)	Bipolar disorder	NONMEM	One	Weight, CrCl, total daily dose, sex	Cl/F (L/h) V1 (L) Ka * (h^−1^)	0.909 × (TDD/600)^0.354^ × (WT/62)^0.33^ × (CrCl/116)^0.186^ 10.9 0.293	20.8 40.4	0.0236 mmol/L

* Fixed value from the literature.

**Table 2:** Characteristics of the external evaluation datasets.

Characteristics	Number or Mean ± SD Clinical	Median (Range) Clinical	Number or Mean ± SD Literature	Median (Range) Literature
No. of patients (male/female)	46 (20/26)	NA	89 (62/27)	NA
No. of samples	46	NA	89	NA
No. of samples per patient	1	NA	1	NA
Age (years)	41.15 (±14.78)	42 (16–68)	24.76 (±14.68)	19 (8–87)
Weight (kg)	74.99 (±18.40)	71.50 (61–127)	66.30 (±22.94)	65 (27–125)
Height (cm)	170.39 (±−10.32)	170 (147–189)	168 (NA)	168 (NA)
Concentration (mg/L)	5.04 (±1.99)	5.14 (1.04–11.94)	4.50 (±2.31)	4.16 (0.69–13.9)
GFR (mL/min)	99.80 (±16.05)	102 (61–127)	120.61 (±88.54)	105.50 (66–218)
Lithium dose (mg)	13.23 (±7.57)	16.22 (3.38–54.05)	15.84 (±8.24)	12.16 (4.05–32.43)
Bipolar patients	46	NA	65	NA

NA: non available.

**Table 3:** Results of the external evaluation for 10 published popPK models (MDPE/MADPE in %).

	MDPE (Literature)	MADPE (Literature)	MDPE (Hospital)	MADPE (Hospital)
Swann et al.	−16.7	29.5	−13.5	26.7
Landersdorfer et al.	−50.9	52.4	−30.2	35.8
Yoshida et al.	17.4	37.4	−4.5	30.5
Eldesoky et al.	99.8	99.8	137.6	137.6
Yu et al.	−85.7	85.7	−42.7	44.2
Couffignal et al.	−8.7	31.5	−4.5	29.9
Methaneethorne et al.	−36.5	40.4	−14.4	24.9
Alqahtani et al.	−5.5	24.4	7.8	32.4
Yuan et al.	−58.4	59.7	−70.0	70.0
Jin et al.	12.7	47.9	18.6	28.7

## Data Availability

The data that support the findings of this study are available from the corresponding author upon reasonable request. The data are not publicly available due to privacy regulation.

## Supplementary Materials

The following supporting information can be downloaded at: https://www.mdpi.com/article/10.3390/ph16111627/s1, Figure S1: Prediction errors (%) versus GFR (mL/min) of the 10 published popPK models on the literature dataset., Figure S2: Prediction errors (%) versus GFR (mL/min) of the 10 published popPK models on the clinical dataset.
