# Supplementary material for: External Evaluation of Population Pharmacokinetics Models of Lithium in the Bipolar Population
Source: Pharmaceuticals (Basel). 2023 Nov 18;16(11):1627. doi: 10.3390/ph16111627 (PMC10674621; doi:10.3390/ph16111627)
Supplement: Supplementary file 1 [file pharmaceuticals-16-01627-s001.zip › pharmaceuticals-2680846-supplementary.pdf]

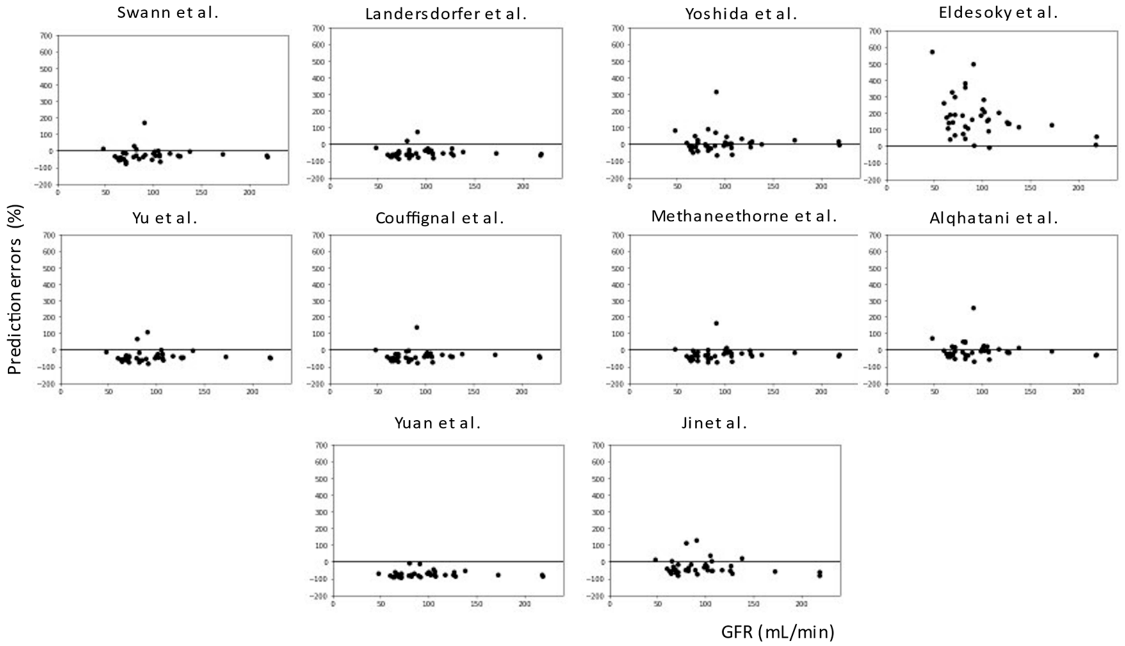

Supplementary **Figure S1.** Prediction errors (%) versus GFR (mL/min) of the 10 published popPK models on the literature dataset.

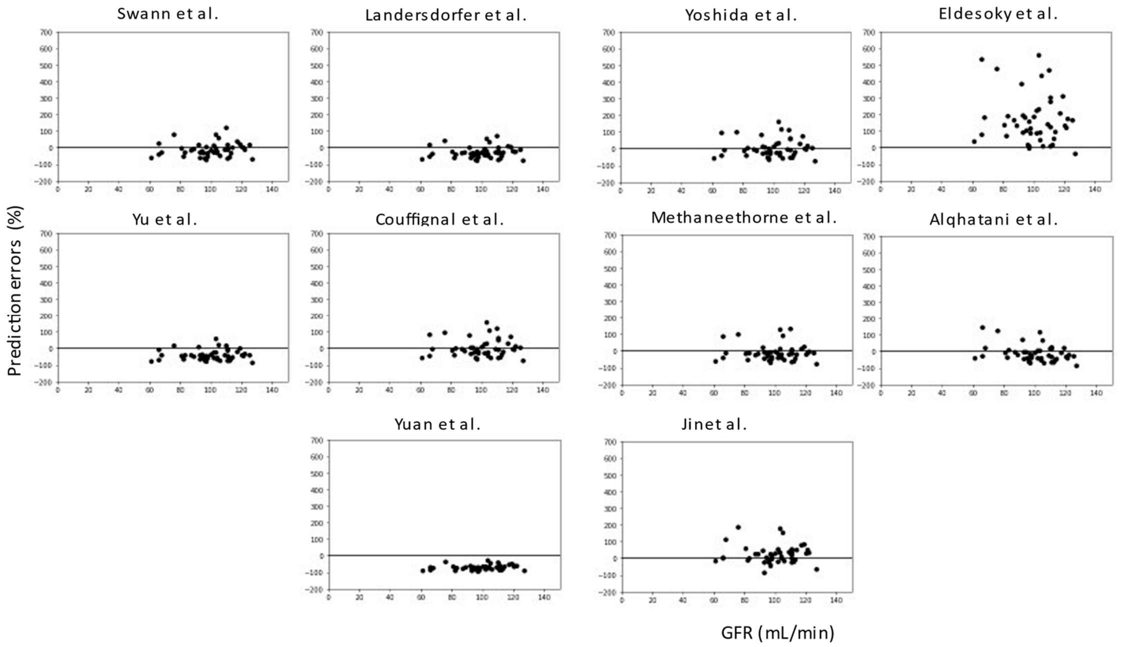

Supplementary **Figure S2.** Prediction errors (%) versus GFR (mL/min) of the 10 published popPK models on the clinical dataset.
